# Physiology and Its Uses

**Published:** 1888-04-14

**Authors:** 


### Physiology and its Uses.

Men have always been making attempts to define man. The priest defines him from one standpoint, the prophet from another, and the metaphysician from a third. Since the birth of the new physiology its followers have claimed their turn, and have defined man from the point of view of that science. These last affirm great scientific authority for their definition, on the ground that they confine their reasonings exclusively to the domain of facts. By facts they mean things which can be taken account of by one or more of the senses; things which can be seen, or heard, or felt, or weighed, or measured, or at least dealt with mathematically. These classes of facts it is said there can be no doubt about: the states of consciousness which constitute the subject matter of the metaphysician and the religious teacher many physiologists hold we are less sure of—so little sure of indeed that it is practically impossible for us to deal with them scientifically at all. This position of the physiologists, the metaphysicians and religious teachers vigorously assail. They affirm, and with perfect justice, that the facts of consciousness are quite as real, quite as capable of being observed and reasoned about scientifically as any other facts. The difference is that they are observed by the mind without the intervention of the senses; whereas physical facts can only be taken account of by one or more of the senses.

We need not attempt here to settle this interminable quarrel. So far as our judgment helps us, the explanation of the difference of opinion seems to be that the typically perfect man is so great, so complex, and so wonderful a being that it requires an intellect of unusual breadth, strength, and subtlety to comprehend him fully. Many able men do not possess this strong and many-sided intellect, but a strictly limited mental capacity. Each of them spends his thinking energy upon that part of man which he sees most clearly, and thus one becomes a metaphysician, another a theologian, and a third a physiologist. Whilst it is comparatively easy for any man of average intelligence and industry to become a schoolmaster, a clergyman, or a physiologist, it takes centuries of time and tens of millions of human beings to produce a Bacon or a Shakespeare.

The physiological man may be approached from two sides—the physical and the mental. He is a material organism; he is also a thinking being. No presentation of him can pretend to completeness which leaves out any portion of his life-history. The physiologist invariably approaches him from the physical side, and for the sake of convenience we may here do the same.

Everybody who is at all likely to read this paper has seen a watch, and knows perfectly well what its uses are. Not only so, but we all know how to take care of it, and what to do to make it continue to serve the purpose for which it was made. That is really all we need ever know about it in ordinary circumstances. For if the watch should at any time go wrong we have the watchmaker at hand who will quickly bring it back to its duty.

But it is possible to imagine a man wishing to have the daily use of his watch, say in Africa, where no watchmaker can be found. In that case, a little knowledge of its structure and mechanism might be of the greatest possible value—might indeed make all the difference between a watch "going" and a watch "stopped" and useless. This remark applies with much force to a little knowledge of physiology. So long as we remain in a civilised country, so long do we possess sufficient knowledge to go on comfortably with our daily duties by the occasional help of the doctor. But if fortune should send us to any outlying region where a doctor could not be obtained, how thankful we might be for a little trustworthy knowledge, however small, about our own material organism.

There is, however, another reason, which some regard as much higher than mere utility, why we should acquire an intelligent knowledge of human physiology. Our bodies and minds are ourselves. All kinds of profound questions are raised in these days by those whose principal study lies in the fields of physiological inquiry. Without some knowledge of the material structure of man, not only the metaphysician but the ordinary reader, who cannot remain outside the influence of the energetic thought-currents of his time, may often be not only intellectually nonplussed, but his moral standing ground may be made to tremble under his feet.

Yet a third plea for the study of physiology is worthy of consideration. It is a region of wonders; a domain of miracles, using the word miracle in the sense of "a sign," or something to be specially noted. The body and the mind; the bony framework and all that it carries about; the limbs and their movements; the heart and its tributaries and effluents; the brain and its messengers; the digestive apparatus and its work; the eye with its power of vision; the ear, with its matchless skill in sound—every organ and every function is a wonder, an inexplicable mystery, a daily miracle. If there were nothing else in the whole world of which man could take intelligent cognisance, the human creature alone, with all that appertains to him, would furnish work for every student the schools of philosophers could produce. Man is the battle-ground for all intellectual soldiers. He has been exalted, he has been abased; yesterday a divinity, to-day an ape; by some regarded as a mere animal, whose brief span of life is shorter than the elephant's, and whose mental powers are a little higher than those of the dog or the chimpanzee; by others claimed as a breath of God, a spark of the immortal fire; an intellectual king and ruler whose kingdom, like that of the Infinite, is destined to be an everlasting kingdom, and whose final state is one of assured victory and honour. "On earth," said one of the profoundest of all thinkers—"on earth there is nothing great but man; in man there is nothing great but mind."

In these articles an attempt will be made to study man as man; not merely the man of the metaphysician, or the man of the physiologist, but the whole, round, complete, and various man as he presented himself to Shakespeare, and as he presents himself to every one who refuses to live enclosed in mental prison walls of any kind, either physical or metaphysical. Physiology, it seems to us, is a science of the deepest possible interest, and one which can be put to the most important uses. It is the writer's wish, and will be his constant effort, to make his readers see its interest and importance as clearly as he sees them.

(*To be continued.*)

